# Human chorionic gonadotropin attenuates amyloid-β plaques induced by streptozotocin in the rat brain by affecting cytochrome c-ir neuron density

**DOI:** 10.22038/ijbms.2018.31412.7569

**Published:** 2019-02

**Authors:** Emsehgol Nikmahzar, Mehrdad Jahanshahi, Leila Elyasi, Mohsen Saeidi, Fatemeh Babakordi, Gozal Bahlakeh

**Affiliations:** 1 Neuroscience Research Center, Golestan University of Medical Sciences, Gorgan, Iran; 2 Neuroscience Research Center, Department of Anatomy, Faculty of Medicine, Golestan University of Medical Sciences, Gorgan, Iran; 3 Stem Cell Research Center, Department of Immunology, Faculty of Medicine, Golestan University of Medical Sciences, Gorgan, Iran

**Keywords:** Amyloid plaque, Brain, Cytochrome c, Human chorionic – gonadotropin, Rat, Streptozotocin

## Abstract

**Objective(s):**

Amyloid β plaques, in Alzheimer’s disease, are deposits in different areas of the brain such as prefrontal cortex, molecular layer of the cerebellum, and the hippocampal formation. Amyloid β aggregates lead to the release of cytochrome c and finally neuronal cell death in brain tissue. hCG has critical roles in brain development, neuron differentiation, and function. Therefore, we investigated the effect of hCG on the density of the congophilic Aβ plaque and cytochrome c-ir neurons in the hippocampus, prefrontal cortex, and cerebellum of Streptozotocin (STZ)-treated rats.

**Materials and Methods:**

Alzheimer model in rats (except the control group) was induced by streptozotocin (3 mg/kg, Intracerebroventricularly (ICV)). Experimental group rats received streptozotocin and then different doses of hCG (50, 100, and 200 IU, intraperitoneally) for 3 days. 48 hr after last drug injection and after histological processing, the brain sections were stained by congo red for congophilic amyloid β plaques and cytochrome c in the hippocampus, prefrontal cortex, and cerebellum were immunohistochemically stained.

**Results:**

Density of congophilic Aβ plaques and cytochrome c-immunoreactive neurons was significantly higher in ICV STZ treated rats than controls. Treatment with three doses of hCG significantly decreased the density of congophilic Aβ plaques and cytochrome c-immunoreactive neurons in the rat hippocampus, prefrontal cortex, and cerebellum in ICV STZ-treated rats (*P*<0.05).

**Conclusion:**

hCG can be useful in AD patients to prevent the congophilic Aβ plaque formation and decrease cytochrome c-immunoreactive neuron density in the brain.

## Introduction

Alzheimer’s disease (AD) accompanies age and over 90 percent of these patients are first diagnosed after age 65 (1). AD causes a progressive loss of cognitive functions (2). Also, AD is becoming an epidemic as well as an economic problem worldwide (3). It is described by the presence of amyloid β (Aβ) plaques, neurofibrillary tangles, neuroinflammation, and oxidative stress (2-4). AD starts in the hippocampus and entorhinal cortex and then affects other parts of the brain cortex (5) and the cerebellum (6, 7). Also in this disease, brain atrophy and ongoing loss of neurons is observed mainly in the frontal cortex, hippocampus, and limbic areas (8).

In AD, Aβ plaques deposit in the hippocampus, amygdala, insular, entorhinal and cingulate cortices, subcortical nuclei, brainstem structures, and the molecular layer of the cerebellum (9). Aβ becomes neurotoxic when it aggregates, and these aggregates lead to a synaptic dysfunction and neuronal cell death in the brain tissue (10-12). One of the important reasons of AD pathophysiology is mitochondrial damage in neurons (3). Additional, increase of Aβ peptide leads to dysfunctioning of mitochondrial Ca^2+^ channels, opening of mitochondrial permeability transition pore (13), which enhances the release of proapoptotic proteins such as cytochrome c and apoptosis-inducing factor from the mitochondria (14, 15). Cytochrome c is a protein found in the inner membrane of the mitochondrion and it participates in the initiation of mitochondrial apoptotic pathways. Transfer of cytochrome c from mitochondria to the cytosol causes the activation of the caspase cascade (15). Hence, inhibition of Aβ accumulation or its effects may be a key solution to inhibit the initiation of AD or slow down its etiological progression (11).

Streptozotocin (STZ), a glucosamine-nitrosourea complex with beta-cytotoxic action, is widely used to prompt oxidative damage, damage glucose metabolism, apoptosis, and tau/Aβ pathology, finally leading to cognitive deficits in both *in vivo* and *in vitro* models of AD (1, 16-18).

Human chorionic gonadotropin (hCG) is a heterodimeric glycoprotein hormone that binds to the G-protein-coupled receptor often called luteinizing hormone (LH)/hCG receptor (19). LH and hCG have a similar structure and share the same receptor, LH/hCG receptor (20). LH/hCG have critical roles in brain development, neuron differentiation, and function (21). In the existence of highly purified hCG, cultured rat neurons have been shown to respond in a dose-dependent manner by growing the neuritic processes and total cellular protein and by decreasing apoptosis (21). hCG has protective effects against oxidative stress through inhibition of apoptosis, activation of cell survival signaling, and keeping mitochondrial function (22). A previous study reported increases in brain Aβ levels after administration of hCG in female rats (23). But in another study on a mouse model of AD, Barron *et al.* (24) showed that treatment by hCG did not change Aβ42 levels. Further, ablation of LH decreases Aβ deposition in Alzheimer amyloid protein precursor (APP) transgenic mice (25). So, the effects of hCG on Aβ levels are contradictory and also, the effects of this hormone on the formation of Aβ plaques and density of cytochrome c-immunoreactive (ir) neurons in Intracerebroventricular (ICV)-STZ rat model of AD is not fully understood. Therefore, we investigated the effect of administration of hCG on the density of congophilic Aβ plaque and cytochrome c-ir neurons in the hippocampus, prefrontal cortex, and cerebellum of STZ-treated rats. 

## Materials and Methods

***Animals***

The experimental protocol was conducted in accordance with the National Institutes of Health Guide for the Care and Use of Laboratory Animals (NIH Publications No. 8023, revised 1978) and all procedures had the approval of the Ethics Committee of Golestan University of Medical Sciences, Gorgan, Iran. The present study was performed on forty Wistar adult male rats weighing 180–220 g (Pasteur Institute, Tehran, Iran), randomly divided into five groups (eight rats in each group). The rats were maintained under a 12-hr light/dark cycle at 22±3 ^°^C while food and water were available *ad libitum*.

**Figure 1:**
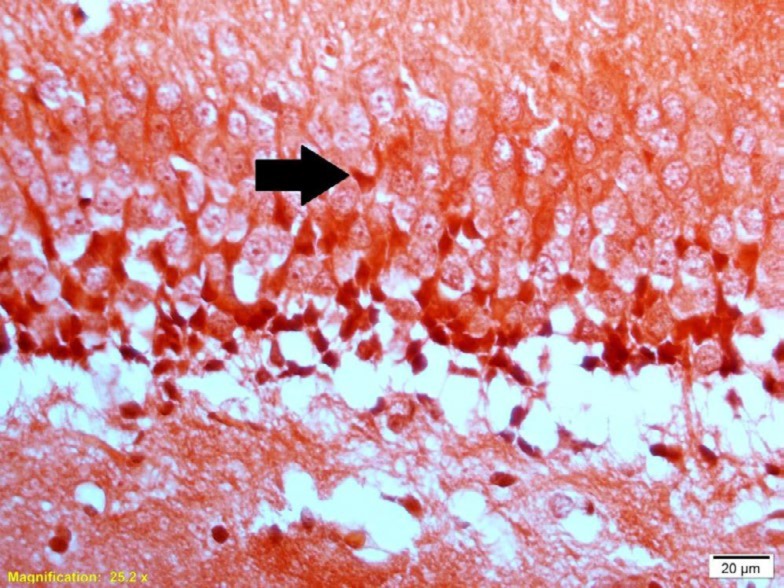
Photomicrograph showing the congophilic Aβ plaques in the DG area of the hippocampus in the STZ + Saline group

Congophilic Aβ plaque is red in color (black arrow). STZ: Streptozotocin; Scale bar is 20 µm

**Figure 2:**
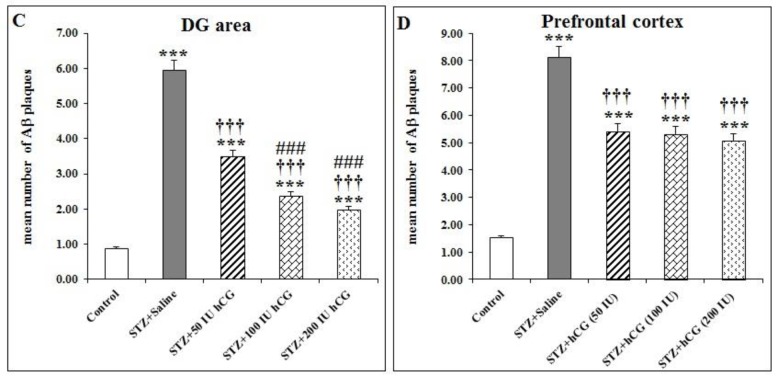
Images of sagittal sections in the prefrontal cortex (A) and cerebellum (B) of STZ induced AD model rats stained by immunohistochemistry with an antibody against cytochrome c. Cytochrome c-ir neuron is brown in color (black arrows)

STZ: Streptozotocin; AD: Alzheimer’s disease; Scale bars are 20 µm for the prefrontal cortex and 10 µm for the cerebellum

**Figure 3:**
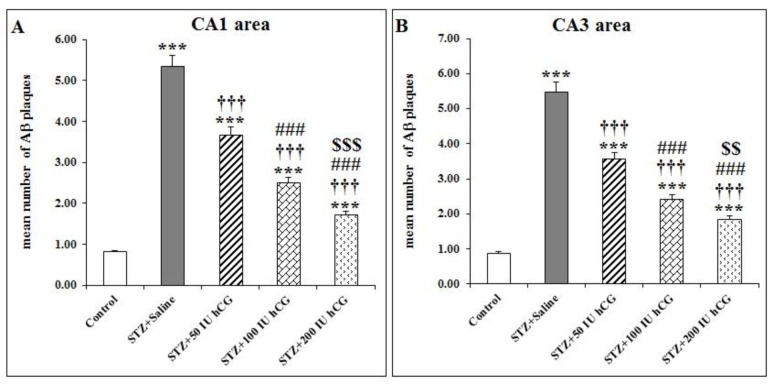
Number of congophilic Aβ plaques after hCG administration in the CA1 (A), CA3 (B), and DG (C) areas of the hippocampus, prefrontal cortex (D), and cerebellum (E) of STZ induced AD model rats

STZ: Streptozotocin; AD: Alzheimer’s disease; Data expressed as mean±SD. *** *P*<0.001 as compared to the control group; ††† *P*<0.001 as compared to the STZ-saline group; ### *P*<0.001 as compared to the STZ+50 IU hCG group; $$ *P*<0.01 and $$$ *P*<0.001 as compared to the STZ+100 IU hCG group

**Table 1:** hCG effect on the number of cytochrome c-ir neurons in the CA1, CA3, and DG areas of the hippocampus in STZ induced AD model rats. Data expressed as mean ± SD

**Areas**	**CA**_1_ ** area (30000 ** **μ** **m** ^2^ **)**	**CA**_3_ ** area (30000 ** **μ** **m** ^2^ **)**	**DG area (30000 μm** ^2^ **)**
**Groups**			
Control	3.12 ± 0.354	1.75 ± 0.707	3.25 ± 0.886
STZ + Saline	11.50 ± 2.070 ***	8.88 ± 1.727 ***	19.12 ± 3.720 ***
STZ+50 IU hCG	6.88 ± 1.126 *** †††	5.25 ± 1.035 *** †††	7.25 ± 0.886 *** †††
STZ+100 IU hCG	5.00 ± 1.512 ** ††† ##	4.83 ± 0.983 *** †††	7.00 ± 1.195 *** †††
STZ+200 IU hCG	3.62 ± 0.744 ††† ### $	3.75 ± 0.707 ** ††† #	5.38 ± 1.302 * †††

* *P*<0.05,

** *P*<0.01, and

*** *P*<0.001 as compared to the control group;

††† *P*<0.001 as compared to the STZ-saline group;

# *P*<0.05,

## *P*<0.01, and

### *P*<0.001 as compared to the STZ+50 IU hCG group;

$ *P*<0.05 as compared to the STZ+100 IU hCG group

**Table 2:** The number of cytochrome c-ir neurons in prefrontal cortex and cerebellum after administration of hCG in STZ-treated rats. Data expressed as mean ± SD

**Areas**	**Prefrontal cortex (30000 ** **μ** **m** ^2^ **)**	**Cerebellum (4800 ** **μ** **m** ^2^ **)**
**Groups**		
Control	1.50 ± 1.414	2.50 ± 0.756
STZ + Saline	6.50 ± 1.309 ***	15.88 ± 2.357 ***
STZ+50 IU hCG	3.75 ± 0.886 *** †††	6.12 ± 1.126 *** †††
STZ+100 IU hCG	3.00 ± 0.926 ** †††	5.75 ± 0.707 *** †††
STZ+200 IU hCG	2.25 ± 0.707 ††† ##	4.12 ± 0.991 * ††† ## $

* *P*<0.05,

** *P*<0.01, and

*** *P*<0.001 as compared to the control group;

††† *P*<0.001 as compared to the STZ-saline group;

## *P*<0.01 as compared to the STZ+50 IU hCG group;

$ *P*<0.05 as compared to the STZ+100 IU hCG group

***ICV injection of streptozotocin***

Adult male Wistar rats were anesthetized with a mixture of ketamine and xylazine (IP) and placed in a stereotaxic apparatus (David Kopf Instruments, USA).

The stereotaxic coordinates for lateral ventricles were −0.8 mm posterior to the bregma, ±1.5 mm lateral, and −4.2 mm deep from the dural surface (26). Under sterile conditions and anesthesia, 21-gauge guide cannulae were implanted in the right and left lateral ventricles. Cannulas were secured with dental cement and acrylate to the skull. Afterward, 27-gauge stainless steel stylets were inserted into the guide cannulae to keep patency prior to microinfusions. The skin was closed with single stitches. Next, the rats were placed in single cages and were permitted to recover for at least 7 days (27, 28). STZ (Sigma, USA) ICV microinjections at a dose of 3 mg/kg in saline (5 μl/injection site) were done on days one and three of the experiment (29) using a 27-gauge injection needle connected to a Hamilton microsyringe (10 µl) by polyethylene tubing.

***Drug administration***

Rats were randomly divided into five groups (eight rats per group) as follows: control, sham (STZ + Saline) and three experimental (STZ+hCG) groups (eight rats per group). Control group rats were the intact group (without surgery and no drug injection). Rats in sham and experimental groups first received ICV injections of STZ (on days one and three of the experiment). Then, 6 days after last ICV injection of STZ, these rats (except the control group) received IP injections of the vehicle (saline, 200 μl) and/or hCG (Darou Pakhsh Pharmaceutical Mfg Co, Iran) with three doses of 50, 100, and 200 IU/200 μl saline (30), for 3 days. The IP injections of hCG and/or saline were given at 9:00 AM.

***Histological examination***

*Tissue preparation:* Forty-eight hr after the last drug injection, the brain tissue was removed by decapitation in deep anesthesia and the brain tissues were fixed in 4% paraformaldehyde for 7 days. Histological processing was done with an automated tissue processing machine (Did Sabz, Urmia, Iran) (31). Serial 6-μm sagittal slices were collected from the hippocampus, prefrontal cortex, and cerebellum (26). Finally, the brain slices were distributed into two sets for different staining separately.

*Congo red staining*

For detection of Aβ plaque deposits in the brain tissue, the brain sections were stained with Congo red (Tek-Path, Turkey) (32, 33). Deparaffinized and hydrated brain sections were incubated with 1% Congo red solution at 56 ^°^C for 45 min. After being washed in saturated aqueous lithium carbonate for 15 sec, slides were stained with Mayer’s hematoxylin solution for 1 min. Then, the slides were dehydrated in 96% and 100% ethanol and cleared in xylene for 3-4 min and coverslipped with Entellan (Merck, Germany) (Figure 1).

*Immunohistochemical staining*

Cytochrome c-ir neurons were evaluated by immunohistochemical staining in the rat hippocampus, prefrontal cortex, and cerebellum according to our previous study (28). Briefly, after deparaffinization and rehydration, retrieval solution (pH=9, Tashkis Baft Arajen, Iran) was used for antigen retrieval at 90–95 ^°^C for 20 min, the slices were allowed to cool at room temperature and then washed with washing buffer (PBS/ Tween 20 in 0.1 % Triton X-100). The activity of endogenous peroxidase was blocked by incubating the brain sections in 0.3% hydrogen peroxide solution in PBS for 10 min at room temperature and washed in washing buffer. For 20 min at room temperature, the sections were blocked with avidin/biotin blocking solution (Dako, Denmark) and then rinsed with washing buffer. Nonspecific reactivity was blocked by adding 1% bovine serum albumin intended for 60 min at 37 ^°^C. Next, the brain sections were incubated with anti-cytochrome c Rabbit polyclonal antibody (1:100, Abcam Inc., USA) for 60 min at 37 ^°^C and rinsed with washing buffer. The secondary antibody was biotinylated goat anti-rabbit IgG (ready to use, Abcam Inc., USA) which was applied to the sections for 60 min at 37^ °^C and then rinsed with the washing buffer. The brain sections were then incubated with streptavidin-HRP protein (1:5000, Abcam Inc., USA) for 30 min at room temperature, and the brain sections rinsed with washing buffer. Specific labeling was visualized using DAB (Dako, Denmark) as chromogen and Meyer’s Hematoxylin was used to stain the background by applying it lightly on the sections for 3–4 sec. To finish, the brain sections were dehydrated in ethanol, cleared in xylene, and mounted with Entellan glue (Merck, Germany) (Figure 2).

*Image processing and cell counting*

Images were captured by an Olympus DP73 digital camera (Japan) equipped with an Olympus BX 53 light microscope (Japan) at a 40× magnification for prefrontal cortex and hippocampal CA1, CA3, and DG areas, at a 100× magnification for the cerebellum. Cytochrome c-ir neurons and congophilic Aβ plaques were counted in a 30000 μm^2^ area at the II / III layers of the prefrontal cortex (34) and in pyramidal layers of CA1 and CA3 areas of the hippocampus and also in the granular layer of hippocampal DG area (27, 28, 35, 36). In the cerebellum, congophilic Aβ plaques were counted in a 30000 μm^2 ^at the molecular layer of the cerebellum (37-39) and cytochrome c-ir neurons in a 4800 μm^2 ^at the granular layer of the cerebellum. All counting for cytochrome c-ir neurons and congophilic Aβ plaques were done by the ImageJ software and imaging and counting were performed blind to treatment.

***Statistical analysis***

Statistical evaluations were carried out using One-way analysis of variance (ANOVA) with *post hoc* LSD test via SPSS software v.16 (Armonk, NY, USA). For all comparisons, the data are presented as means±SD. *P*<0.05 was considered significant.

## Results

***hCG effect on congophilic Aβ plaque density in brains of ICV STZ-treated rats***

One-way ANOVA analysis and LSD *post hoc* test demonstrated that density of congophilic Aβ plaques in CA1, CA3, and DG areas of the hippocampus were significantly higher in the STZ+Saline group compared with the control group (*P*<0.001; Figures 3A–C). 

There was significant difference of congophilic Aβ plaque density in the prefrontal cortex between STZ+Saline and control groups (*P*<0.001; Figures 3D, E).

Also, the difference of congophilic Aβ plaque density in the cerebellum between STZ+Saline and control groups was significant (*P*<0.001; Figures 3D, E). 

After treatment with usage of different doses of hCG, the concentration of Aβ plaque was decreased in all hippocampal sub-regions. These differences were significant as compared with the STZ+Saline group (*P*<0.001; Figures 3A–C).

In CA1, CA3, and DG sub-regions of the hippocampus, administration of hCG with 100 and 200 IU could significantly decrease the congophilic Aβ plaque density as compared with the STZ+50 IU hCG group (*P*<0.001; Figures 3A, B). 

Also, in CA1 and CA3 areas of the hippocampus, the congophilic Aβ plaque density was significantly different between STZ+100 IU hCG and STZ+200 IU hCG groups (*P*<0.001 and *P*<0.01, respectively; Figures 3A, B). 

There was significant decrease in congophilic Aβ plaque density as compared with the STZ-saline group in the cerebellum and prefrontal cortex after administration of hCG with three doses (*P*<0.001; Figures 3D, E). But in the cerebellum and prefrontal cortex, after treatment with hCG at doses of 50, 100, and 200 IU, significant differences between those groups was not observed.

***hCG effect on the density of cytochrome c-ir neurons in brains of ICV STZ-treated rats***

Tables 1 and 2 show that in the STZ+Saline group, ICV injection of STZ significantly increased the number of cytochrome c-ir neurons in comparison with the control group in all areas of the hippocampus, prefrontal cortex, and cerebellum (*P*<0.001; Tables 1, 2). 

Treatment with hCG (doses 50, 100, and 200 IU, IP) for three days significantly decreased cytochrome c-ir neuron density in all areas of hippocampal formation in comparison to the STZ+Saline group (*P*<0.001; Table 1).

In CA1 area of the hippocampus, injection of 100 IU hCG could significantly decrease cytochrome c-ir neuron density in comparison with the STZ+50 IU hCG group (*P*<0.01; Table 1). Also in this area, significant difference was observed between STZ+100 IU hCG and STZ+200 IU hCG groups in cytochrome c-ir neuron density (*P*<0.05; Table 1).

In CA1 and CA3 areas of the hippocampus, cytochrome c-ir neuron density in the STZ+200 IU hCG group was statistically lower compared with the STZ+50 IU hCG group (*P*<0.001 and *P*<0.05, respectively; Table 1).

In DG area of the hippocampus, there was no significant difference between the hCG-treated groups in cytochrome c-ir neuron density.

Also, hCG administration at doses of 50, 100, and 200 IU (IP, for three days) decreased the density of cytochrome c-ir neurons in the prefrontal cortex and cerebellum and this decrease was statistically significant (*P*<0.001 compared with the STZ+Saline group; Table 2).

In the prefrontal cortex and cerebellum, treatment with hCG at the dose of 200 IU reduced the density of cytochrome c-ir neurons significantly as compared with the STZ+50 IU hCG group (*P*<0.01; Table 2). In the cerebellum, we also observed significant differences between STZ+100 IU hCG and STZ+200 IU hCG groups (*P*<0.05; Table 2).

## Discussion

Administration of hCG (50, 100, and 200 IU) to STZ-treated rats significantly decreased the density of congophilic Aβ plaques and cytochrome c-ir neurons in the hippocampus, cerebellum, and prefrontal cortex of rat brains. These effects were dose-dependent, so we found most effects in the high dose of hCG.

Extracellular amyloid plaques are one of the major histopathological hallmarks of AD (40), and mostly deposited in the brain cortex, in the hippocampus (2), in the molecular layer of the cerebellum, and rarely in the granule cell layer of the cerebellum (37, 38). Many features of AD were observed following ICV injections of STZ in rodents (41, 42), such as accumulation of Aβ (41, 43) in the hippocampus (42, 44). We also found an accumulation of congophilic Aβ plaques in the hippocampus, prefrontal cortex, and cerebellum after ICV injections of STZ.

Some previous studies showed that hCG administration leads to an increase in Aβ40 accumulation in a mouse model of AD (23, 24, 45). But Barron *et al.* reported that levels of Aβ42 did not change with hCG treatment in a mouse model of AD (24), while administration of hCG to ovariectomized rats increased soluble Ab1-40 and Ab1-42 levels (23). Indeed, hCG promotes the amyloidogenic pathway of APP metabolism in which Aβ is formed (45, 46). Also, adding of hCG to human embryonic stem cells can cause increased expression in all forms of Aβ precursor protein (47), while SHSY5Y neuroblastoma cells increased β-cleavage of the amyloid precursor protein when treated to high levels of hCG (45). Several studies have reported that LH ablation and also genetic ablation of the LH receptor can lead to reduction of Aβ plaques in the hippocampus and cerebral cortex in a mouse model of AD (25, 48).

It is documented that mitochondria play a major role in the regulation of cell death, particularly cell apoptosis and mitochondrial dysfunction are symbols of Aβ-induced toxicity of neurons in AD (49). The mitochondria-mediated cell death was evaluated by measuring the release of cytochrome c into the cytosol and following activation of caspase-3 (50).  Indeed, the release of cytochrome c is the first step in an apoptotic pathway (51, 52). Also, STZ-ICV administration in rats induces mitochondrial abnormalities in rat brains (44). It is reported that STZ exposure of cultured neurons can cause significant disturbance of glucose uptake and mitochondrial function that then results in mitochondrial membrane potential damage, excessive calcium overload (53), translocation of cytochrome c in the cytosol, increased appearance of caspase-3, DNA damage, and finally neuronal death (54). Our data indicated that ICV injection of STZ to rats causes increased cytochrome c-ir neuron density in the brain. 

It seems release of cytochrome c from mitochondria to the cytosol can increase by STZ-induced Aβ deposition. Indeed, Aβ can induce mitochondrial dysfunction and also lead to the release of cytochrome c (55, 56); an apoptosome is formed and it activates the initiating protease caspase-9, which in turn activates the executioner caspases-3, and finally results in apoptosis (57, 58). In our present study, ICV injection of STZ to rats increased the density of congophilic Aβ plaques and cytochrome c-ir neurons in the hippocampus, prefrontal cortex, and cerebellum, this increase was statistically significant. 

Indeed, it was reported that treatment with hCG can stimulate the proliferation of cultured human ovarian surface epithelium cells, and it can inhibit the apoptosis of these cells induced by serum deprivation. The signaling of LH/hCG followed by up-regulation of insulin-like growth factor-1 is presumably elaborated in the inhibition of apoptosis of human ovarian surface epithelium cells (59). Also, the proliferation of endogenous neural stem cells is promoted by hCG (60). The previous results indicated that gonadotropins, such as hCG, have the potential to induce cell proliferation and protect from cell death *in vitro* (61, 62). Likewise, by pretreatment with hCG, cytochrome c, p-Bax, p-caspase 9, and caspase 3 activation was decreased in comparison with that found in the absence of hCG. Moreover, these effects were accompanied by raised activation of extracellular-signal-regulated kinases 1/2 and Akt (22).

In summary, we found that treatment with hCG can decrease the rise in density of congophilic Aβ plaques and cytochrome c-ir neurons in rat brains. These results showed that the protective effect of hCG in attenuating STZ-induced congophilic Aβ plaques can increase through reduction of cytochrome c-ir neuron density.

## Conclusion

We concluded that hCG can be useful for the treatment of dementia and Alzheimer’s disease by preventing the formation of congophilic Aβ plaques and decreasing cytochrome c-ir neuron density in the brain of AD patients. It seems hCG can decrease the congophilic Aβ plaque formation by decreasing cytochrome c-ir neuron density in the ICV STZ-treated rat brains.
